# Scientific laws of research funding to support citations and diffusion of knowledge in life science

**DOI:** 10.1007/s11192-022-04300-1

**Published:** 2022-03-05

**Authors:** Melika Mosleh, Saeed Roshani, Mario Coccia

**Affiliations:** 1 Birmingham Business School, College of Social Sciences, University of Birmingham, Birmingham, UK; 2 Department of Technology and Entrepreneurship Management, Faculty of Management and Accounting, Allameh Tabataba’i University, Tehran, Iran; 3 National Research Council of Italy, Collegio Carlo Alberto, Via Real Collegio, Torino, Italy

**Keywords:** Research funding, Research policy, Decision-making matrix, Science diffusion, Scientific development, Power-law distribution

## Abstract

One of the main problems in scientometrics is to explore the factors that affect the growth of citations in publications to identify best practices of research policy to increase the diffusion of scientific research and knowledge in science and society. The principal purpose of this study is to analyze how research funding affects the citation-based performance of scientific output in vital research fields of life science, which is a critical province (area of knowledge) in science to improve the wellbeing of people. This study uses data from the Scopus database in 2015 (to assess the impact on citations in 2021, after more than 5 years) concerning different disciplines of life science, given by “agricultural and biological sciences”, “biochemistry, genetics, and molecular biology”, “Immunology and microbiology”, “neuroscience” and “pharmacology, toxicology and pharmaceutics”. Results demonstrate that although journals publish un-funded articles more than funded publications in all disciplines of life science, the fraction of total citations in funded papers is higher than the share in the total number of publications. In short, funded documents receive more citations than un-funded papers in all research fields of life science under study. Findings also support that citations of total (funded + un-funded), funded, and un-funded published papers have a power-law distribution in all five research fields of life science. Original results here reveal a general property in scientific development: funded research has a higher scaling potential than un-funded publications. Critical implications of research policy, systematized in a decision-making matrix, suggest that R&D investments in “Neuroscience” can generate a positive impact of scientific results in science and society-in terms of citations-higher than other research fields in medicine. Overall, then, results here can explain some characteristics driving scientific change and help policymakers and scholars to allocate resources towards research fields that facilitate the development and diffusion of scientific research and knowledge in life science for positive societal impact.

## Introduction

Several studies in scientometrics have investigated the role of different factors in scientific change, such as public research labs, publications, emerging research fields, nations’ research performance, etc. (Coccia, 2005a, 2005b, 2009, 2019a; Coccia et al., 2015; Pagliaro & Coccia, 2021; Radicchi et al., 2008; Yan et al., 2018; Zhao et al., 2018). A central question in social studies of science is how to enhance the diffusion of scientific research in vital research fields, such as medicine, to increase societal impact. In scientometrics, the scientific performance of papers has been investigated by many scholars, such as Li et al. (2013) that focus on the role of authorship network’s impact on publication performance. Scholars also analyze the effects of institutions’ collaboration on citation performance, including ranking of academic affiliation (Petersen, 2014) or the scaling relationship between journals’ impact and scientific performance (Larivière, 2010; Thelwall & Wilson, 2014). Other studies investigate the impact of multiple funding on citation performance (MacLean, 1998), the impact factor of journals (Campanario et al., 2011), the relationship between public/private funding and scientific production in nanotechnology (Beaudry, 2012), the role of research sponsorship on research performance in nanoscience (Wang & Shapira, 2011), etc. Reed et al. (2007) show that the quality of published research is associated with their funding status in medical education. Gök et al. (2015) suggest that citation impact is associated positively with funding diversification and negatively with funding intensity. Checchi (2019) introduces a performance-based research funding system and analyzes its effect on the quantity and quality of different nations’ publications. Yan et al. (2018) maintain that funded rather than un-funded publications receive more citations. Quinlan et al. (2008) point out that funded publications attract more citations than un-funded documents. Scholars also argue that funding mainly affects the citations, and funded papers can attract more usage, though a variation exists between research fields (Morillo, 2020; Pao, 1991). Zhao et al. (2018) suggest that research funding is an essential resource in science’s reward system. Another study shows that participating in a competition to get the grant regardless of the result can help scientists to accelerate their citations because of their efforts to prepare the proposal, communicate with other authors to get the grant, etc. (Ayoubi et al., 2019). Heyard et al. (2021) analyze the relationship between funding and authors’ altered metrics scores and show that funded studies got more public attention than other studies.

Moreover, Fleming et al. (2019) interestingly investigate the impact of funding on innovation according to patents performance and claim that federal supports accelerate innovation production. In general, the literature suggests that funded research has a more substantial impact than un-funded research in science that is magnified by other factors, such as collaboration or authorship affiliation (Rigby, 2013). Financial resources can accelerate the diffusion of science and new knowledge to support society’s wellbeing (Laudel, 2006; Roshani et al., 2021). Some studies also suggest no significant relationship between funding and publication performance in some areas (Jacob & Lefgren, 2011).

In the presence of inconsistencies and ambiguities in the literature on these topics, the relationship between research funding by agencies and the level of papers’ citations and diffusion of knowledge in the life science research field is hardly known. In fact, life science currently plays a central role in supporting policy responses and drug discovery processes to cope with the COVID-19 pandemic crisis (Coccia, 2021a, 2021b, 2021c, 2021d, 2021e, 2021f). Stimulated by these fundamental problems and gaps in the literature for a vital scientific domain, this study aims to analyze the relationship between the citation-based performance of articles and their funding status in disciplines of life science that play a vital role in improving people’s wellbeing. The assumption behind this study is that we just considered the funded publications by agencies in journals which are recorded in global dataset, showing status concerning funding of papers. This study focuses on the investigation of the relationship between funding and publication performance (measured with citations) in life science to find out, as far as possible, general characteristics of the scientific change that can explain science dynamics and support research policies directed to improve the effectiveness of funding allocation for knowledge creation and diffusion in science and society. This study applies an approach of computational scientometrics (the application of bibliometric and scientometrics methods to large-scale datasets) to analyze these topics and suggest implications of research funding policy. The paper here is part of a large body of research that endeavors to successfully identify the driving factors of the evolution of science and technology to support best practices of research and innovation policy in society (cf., Coccia & Wang, 2016).

## Theoretical background

In order to examine the relationship between funding and citation impact, some studies are reported here to review the current state-of-the-art in these topics. Several studies have investigated different aspects of scientific performance, including the number of citations, publications, word occurrences across documents, collaborations, emerging disciplines, etc. (Peterson, 2010: Coccia et al., 2021). Aksnes et al. (2019) argue that citations are increasingly used as performance indicators within the research system, and scientists with high reputation and many citations will attract more scientific opportunities (e.g., grants, international collaborations, etc.), leading to better scientific performance and further recognition. de Solla-Price (1976) claims that the citation performance of a paper depends on its previous citations. In addition, the highest cited articles appear prior to others in the search engine results, increasing popularity and citations (Aksnes et al., 2019). Scholars show that a scaling relationship can exist between the number of publications and citations (cf., Barabási & Albert, 1999; Ferraro et al., 2009; Merton, 1988; Tahamtan et al., 2016; Glänzel, 2007). Ronda-Pupo and Katz (2018) show the existence of a scaling relationship among scientific resource performance features. Van Raan (2008) analyzes the scaling relationship between citations and field citation density to demonstrate that top performance universities tend to publish more papers in journals with high impact than lower performance universities. Results also suggest that top performance universities have more opportunities to collaborate with other universities, receive more funds, and get popularity among scholars, encouraging the journals to be more likely to publish their scientific results (Coccia & Bozeman, 2016; Hicks & Katz, 2011). Laudel (2006) maintains that researchers rewarded with funding are more likely to receive funds for their future studies. Other studies show that authors who have received grants and a higher level of funding can receive more citations than non-funded papers (Amara et al., 2015). Especially, scientists identify similar patterns in many bibliometric data that lead to the Matthew effect, given by: “the accruing of greater increments of recognition for particular scientific contributions to scientists of considerable repute and the withholding of such recognition from scientists who have not yet made their mark” (Gillespie, 2015; Merton, 1968, 1988; Ye & Rousseau, 2008). In this regard, Roshani et al. (2021) find that combined, funded, and un-funded research citations follow a power-law distribution in computer science, medicine, and economics. Roshani et al. (2021) also show a stronger scaling relationship in funded documents than un-funded papers in the previously mentioned research fields. In short, funded documents received more citations than un-funded papers in computer science, medicine, and economics, and consequently, the Matthew effect is greater for funded articles compared to un-funded documents. Furthermore, papers receiving industry funding can have more citations if their scientific results are pro-industry (Farshad et al., 2013; Kulkarni et al., 2007). Instead, some studies show a low association between citations and grants (Boyack & Börner 2003).

In order to extend this research stream (Roshani et al., 2021), the study here investigates the relationship between funded and un-funded paper counts and the number of citations in life science, as well as the existence of a power-law that can clarify the scientific change of research fields and help policymakers in making efficient decisions regarding sponsoring specific research fields of life science that can foster the scientific development with fruitful effects for the wellbeing of people in society.

## Materials and methods

### Research questions and motivation of the study

The main research questions of this study are:

- How is the relationship between research funding and citations in life science publications?
- Are there any differences between the growth of the citations in funded and un-funded research in life science?
- Which areas of life science are critical to be funded for major knowledge creation and diffusion?
- Does computational scientometrics provide some decision-making information to support funding in life science?

This study examines these questions to explain, whenever possible, the behavior of citation in funded and un-funded articles of life science and how funding can impact on citations for the diffusion of scientific research and knowledge in science and society.

### Sample and sources

This study focuses on research fields of life science, considering data from the Scopus database (Scopus, 2021). Research fields of life science under study are:

- Agricultural and biological sciences.
- Biochemistry, genetics and molecular biology.
- Immunology and microbiology.
- Neuroscience.
- Pharmacology, toxicology, and pharmaceutics.

These five research fields are the most representative and important life science research areas in the Scopus database classification and method of inquiry here is based on a comparative analysis to detect similarity and differences (Coccia & Benati, 2018; Coccia, 2018a). Data are all publications in 2015 for five research fields of life science just mentioned, using the Scopus database having the number of citation counts (Scopus, 2021). The year 2015 is chosen to have a period of more than five years, until 2021, to assess the performance of publications with their citations. The publication types are limited to articles and reviews published in journals. The string search for retrieving agricultural and biological sciences data is SUBJAREA (AGRI), refined by document types: (Articles OR Review). We changed the SUBJAREA value to “BIOC”, “IMMU”, “NEUR”, and “PHAR” to collect data related to the fields of biochemistry, genetics, and molecular biology, immunology and microbiology, the category of neuroscience, and lastly, pharmacology, toxicology, and pharmaceutics.

### Measures

This study analyzes the papers published in 2015, considering a time lag of more than 5 years for assessing citations adequately over time in “agricultural and biological sciences”, “biochemistry, genetics and molecular biology”, “immunology and microbiology”, “neuroscience”, and “pharmacology, toxicology and pharmaceutics”.

Funding variables categorize articles and their citations into two categories:

- Funded articles pertain to articles that are published in journals having a funding for performing the scientific research.
- Un-funded articles are the published articles in journals that had not received any funding.
- Total category is based on funded and un-funded articles.

The number of citations is the main indicator of citation-based performance in these different sets mentioned: funded, un-funded, and the Total (funded and un-funded) articles of disciplines under study (Scopus, 2021).

### Theoretical and empirical strategy and data analysis procedure

The analysis of research questions is developed through three main steps.

Firstly, this study focuses on the power-law distribution of Total citations in (funded + un-funded)/funded/un-funded articles published in “agricultural and biological sciences”, “biochemistry, genetics and molecular biology”, “immunology and microbiology”, “neuroscience”, and “pharmacology, toxicology and pharmaceutics”. The power-law distribution is a suitable model to assess the relationship between the number of publications and their performance with citations according to funding status, as confirmed by other studies (Alstott, et al., 2014; Ronda‐Pupo & Katz, 2016, 2018; Roshani et al., 2021).

Secondly, we measured the power-law correlation between citations and numbers of Total (funded + un-funded)/funded/un-funded publications.

*Power-law distribution verification.* This study applies the procedure by Clauset et al. (2009, p. 3) to analyze the power-law distribution of the citations of Total (funded+un-funded)/funded/un-funded articles. Xmin value and scaling parameter α of the power-law are calculated. Xmin is the number of publications when the power-law scaling starts from this level (Clauset et al., 2009). To measure the Xmin value, we used the formula by Clauset et al. (2009):

$$\widehat{\alpha }\simeq 1+n{\left[\sum_{i=1}^{n}ln\frac{{x}_{i}}{{x}_{\mathrm{min}}-\frac{1}{2}}\right]}^{-1}$$

Throughout this study, we apply the maximum probability method to calculate the parameter α. We calculated the fitting goodness of power-law through the data afterwards. We used the bootstrap function of the Monte-Carlo simulation method with a disturbance rate of 2500 to identify whether the power-law distribution can be a plausible pattern for data in life science. Finally, we calculated the Kolmogorov-Smirnov (KS) statistics to measure the fitness of the power-law model for each sample individually. We calculate, in this step, the *p*-value of the model. In power-law distribution, the *p*-value is consistent whenever it is higher than 0.1, indicating an adequate distribution for the data.

Thirdly, the power-law correlation between the number of citations and the number of (funded+un-funded)/funded/un-funded papers in disciplines of life science is analyzed. Citation-based performance (CBP) relates to all citations obtained by publications. CBP is a response variable in model, whereas the number of papers published in journals is the explanatory variable. The model by Ronda-Pupo and Katz (2016) is applied to test the power-law correlations between citations and Total (funded+un-funded)/funded/un-funded articles published in life science journals:

1 $$CBP \, = \, kc^{\alpha }$$

*CBP* = Citation-Based Performance, *c* = number of articles (total: funded+un-funded, funded and un-funded), *k* = constant, *α*= scaling factor.

Parameter *α* is a proxy of the Matthew effect to show the scaling correlation exponent between articles published in journals and their citations. The Ordinary Least Squares (OLS) method estimates the parameter α and the parameter *k* (Leguendre & Leguendre, 2012). The scaling exponent *α* can be used to predict the behavior of correlation. In particular (Ronda-Pupo, 2016):

- Scaling exponent *α* > 1, then CBP does not scale linearly, and the number of citations increases at a higher rate than the number of published articles in life science journals. Thus, correlations are nonlinear, and we have a positive cumulative advantage.
- Scaling exponent of *α* < 1, then correlation is sub-linear: there is a cumulative disadvantage, which means that the research citations increase slowly compared to the publication rate in journals.
- Scaling exponent of *α* = 1 shows a linear correlation between variables, such that the number of citations and journal publications increase at the same rate of growth.

Afterward, we employed a *t* test to confirm if the scaling exponents of the power-law correlation have statistically a significant level.

**Fig. 1:**
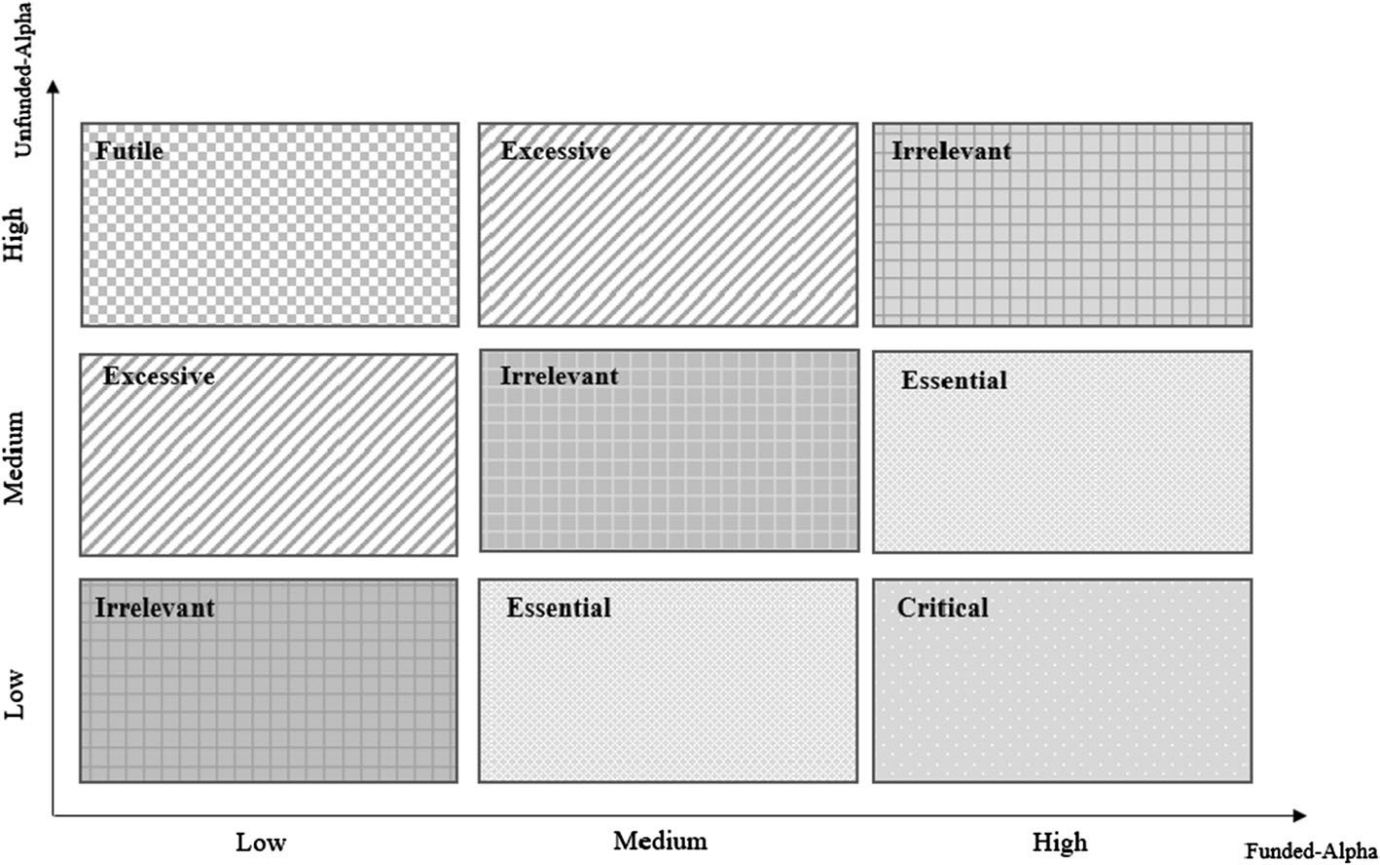
Theoretical decision-making matrix for decision support of funding research policy in scientific fields

Finally, results can be used to suggest a nine-box matrix that is a tool used in research policy to analyze scientific performance in terms of citations of research fields based on funded and/or un-funded publication to support policymakers in R&D investments of promising research fields. This matrix combines two dimensions: the *x*-axis of diagram represents the alpha value of funded articles categorized (with an assumption of equal distribution) in low, medium, and high levels; the *y*-axis represents the alpha value of un-funded publications with the same classification. The relationships between alpha of funded and un-funded papers in funding matrix indicate the strategic positioning of research fields (in boxes or zones) considering the Matthew effect for supporting critical aspects of research funding: Critical, Essential, Irrelevant, Excessive and Futile (Fig. 1).

In this regard, five concepts that can prioritize research funding in different fields are defined considering the alpha of funded articles on the *x*-axis and alpha of un-funded papers on the *y*-axis:

- *Critical* a high gap between alphas of funded and un-funded articles shows that funding might greatly impact citation growth, meaning that financial support policy in this field should be a critical and primary aspect for citation performance improvement.
- *Essential* a medium gap between alphas of funded and un-funded articles; all fields positioned in the essential area should have a secondary priority level in funding policy.
- *Irrelevant* funded and un-funded alphas are in the same section (low and low, medium and medium, or high and high). Funding of these research fields does not support citation; accordingly, funding in the research fields included in this section has a lower priority than previous categories.
- *Excessive* the alpha value of funded publications is in a lower section than un-funded research, showing an inverse relationship between funded and citations performance; empirical analysis here shows that funding cannot facilitate citation growth.
- *Futile* funded publications alpha is in the low section, and un-funded alpha is in the high section; in this case, funding might play an inverse role in citation performance and deteriorate the impact of the publication.

## Results and discussions

Research fields of life science under study here are:

- Agricultural and biological sciences.
- Biochemistry, genetics and molecular biology.
- Immunology and microbiology.
- Neuroscience.
- Pharmacology, toxicology and phsarmaceutics.

We collected documents from the Scopus (2021) database.

- Accordingly, 187,276 documents are studied in agricultural and biological sciences, including articles and reviews, with 3,044,146 citations published in 1,970 journals. 27.79% of documents had been supported financially and received 38.67% of all citations. Moreover, 72.21% of published papers are un-funded but received a smaller proportion of total citations, around 61.33%.
- We also analyzed 282,182 documents with 6,308,179 citations in 2,006 journals of Biochemistry, Genetics and Molecular Biology. In this field of research, 48.90% of the citations account for 36.10% of all documents supported by funding agencies.
- Results also show 65,734 documents in 534 journals of Immunology and Microbiology, in which 46.29% of citations accounted for 33.24% of all documents, which are funded papers.
- The neuroscience research field has 62,323 documents in 578 journals, and 56.94% of its total citations accounted for funded documents (44.86% of all publications).
- Finally, in 717 Pharmacology, Toxicology and Pharmaceutics journals, results reveal that there are 78,360 records and 43.57% of citations accounted for financially supported publications (31.40% of the Total).

Table 1 shows that funded documents in life science journals received more citations than un-funded papers in all five research fields under study, suggesting the beneficial role of sponsorship on the spread of scientific research in this field of research.

**Table 1:** The number of documents and citations in research fields of life science according to funding status

Research fields	Sources	Number of papers	%	Citations	%
Agricultural and biological sciences	Total^a^	187,276	100	3,044,146	100
	Funded	52,048	27.79	1,177,076	38.67
	Un-funded	135,228	72.21	1,867,070	61.33
Biochemistry, genetics and molecular biology	Total^a^	282,182	100	6,308,179	100
	Funded	101,875	36.10	3,084,843	48.90
	Un-funded	180,307	63.90	3,223,336	51.10
Immunology and microbiology	Total^a^	65,734	100	1,477,313	100
	Funded	21,847	33.24	683,797	46.29
	Un-funded	43,887	66.76	793,516	53.71
Neuroscience	Total^a^	62,323	100	1,425,246	100
	Funded	27,956	44.86	811,597	56.94
	Un-funded	34,367	55.14	613,649	43.06
Pharmacology, toxicology and pharmaceutics	Total^a^	78,360	100	1,257,310	100
	Funded	24,600	31.40	547,863	43.57
	Un-funded	53,760	68.60	709,447	56.43

^a^Total: the combination of funded and un-funded publications

Research fields in life science can be categorized in:

- *High intensity of research funding*, such as biochemistry and neuroscience (number of publications > 35%).
- *Medium intensity of research funding,* including immunology and microbiology, and pharmacology (number of publications: 30–34%).
- *Low intensity of research funding* is agricultural and biological sciences (number of publications < 30%).

According to Clauset et al. (2009), Xmin is the value when the power-law begins. α is the scaling factor (slope of the *log*-*log* regression line). The Kolmogorov–Smirnov (KS) statistic is an indicator to demonstrate the distance between the sample’s empirical distribution and the reference’s cumulative distribution function.

This study employed Monte-Carlo bootstrapping analysis by 2500 iterations to fit the citations data to a power-law distribution. Table 2 shows the simulation results for five research fields in Total (funded + un-funded), only funded, and only un-funded documents. The statistical analysis supports the model of power-law distribution for all three *status* of funding in “agricultural and biological sciences”, “biochemistry, genetics and molecular biology”, “immunology and microbiology”, “neuroscience”, and “pharmacology, toxicology and pharmaceutics”. Surprisingly, “pharmacology, toxicology and pharmaceutics” is the only field in which the *X*min parameter of funded papers is lower than un-funded documents; however, the number of un-funded publications, in general, is more. Therefore, financial resources can generate an efficient impact in this field of research. In particular, the funded category needs a lower initial number of citations to start its positive scaling growth. Table 3 shows the comparative analysis between distributions. The *p*-value shows the significance level, and the log-likelihood ratio test (LR) is the main factor in comparing alternative distributions.

**Table 2:** Results of fitting the power-law to the datasets

Research fields	Funding	Xmin	*α*	KS
Agricultural and biological sciences	Total^a^	4.574	2.45***	0.052
	Funded	4.009	2.68***	0.063
	Un-funded	2.880	2.59***	0.038
Biochemistry, genetics and molecular biology	Total^a^	5.626	2.32***	0.043
	Funded	6.435	2.41***	0.052
	Un-funded	3.341	2.43***	0.020
Immunology and microbiology	Total^a^	5.655	2.51***	0.064
	Funded	2.128	2.17***	0.079
	Un-funded	1.631	2.30***	0.056
Neuroscience	Total^a^	8.595	3.12***	0.093
	Funded	4.538	2.57***	0.071
	Un-funded	2.939	3.05***	0.075
Pharmacology, toxicology and pharmaceutics	Total^a^	9.117	3.31***	0.070
	Funded	1.974	2.29***	0.104
	Un-funded	2.159	2.81***	0.071

^a^The combination of funded and un-funded publications

^***^Denotes significance when Power-Law *p*-value > 0.1

**Table 3:** Results of comparing the power-law with alternative distributions

Research fields	Funding	Power-law	Power-law + cut-off	
		*p*	*LR*	*p*
Agricultural and biological sciences	Total^a^	0.29	0.01	0.99
	Funded	0.60	0.001	0.99
	Un-funded	0.85	0.11	0.79
Biochemistry, genetics and molecular biology	Total^a^	0.44	0.01	0.99
	Funded	0.61	− 1.28	0.10
	Un-funded	0.93	− 0.18	0.82
Immunology and Microbiology	Total^a^	0.47	− 1.22	0.11
	Funded	0.16	− 1.71	0.01
	Un-funded	0.33	− 1.17	0.10
Neuroscience	Total^a^	0.13	− 0.43	0.67
	Funded	0.56	− 0.90	0.30
	Un-funded	0.33	− 1.07	0.16
Pharmacology, toxicology and pharmaceutics	Total^a^	0.50	− 1.03	0.16
	Funded	0.01	− 2.01	0.002
	Un-funded	0.85	− 0.21	0.42

^a^Total: the combination of funded and un-funded publications

Table 4 shows that the power-law correlation between the number of published papers and their citation-based performance in Total (funded + un-funded), funded, and un-funded categories in all research fields under study are statistically significant with a *p*-value < 0.001. The results reveal that the Matthew effect is greater in funded documents than un-funded ones in all three status sets of five research fields in life science. Agricultural *and Biological Sciences* show that the number of funded papers grows, scaling the research citation performance. In *Biochemistry, Genetics and Molecular*, the Matthew effect of funded category is greater than un-funded one, such that funding has a considerable impact on scientific research expansion. Though a positive Matthew effect in un-funded papers in journals of Immunology and Microbiology, the scaling level is lower than other categories under study. In *Neuroscience*, the Matthew effect is more significant in funded articles than un-funded ones. In fact, we can expect a higher level of citation growth in this research field by increasing the number of funded documents rather than un-funded research papers. In *Pharmacology, Toxicology and Pharmaceutics*, the Matthew effect of the funded category is more remarkable than other categories. Hence, it can be concluded that a funding strategy can support the research impacts through a strong scaling growth of citation-based performance of papers in life science.

**Table 4:** Values of the exponents for the power-law correlation

Research fields	Funding	α	Δ	(SD)	*R*^2^	*N*
Agricultural and biological sciences	Total^a^	1.35***		(0.01)	0.74	1970
	Funded	1.30***	0.04	(0.01)	0.83	1376
	Un-funded	1.26***		(0.01)	0.67	1960
Biochemistry, genetics and molecular biology	Total^a^	1.26***		(0.01)	0.76	2006
	Funded	1.21***	0.04	(0.01)	0.81	1294
	Un-funded	1.17***		(0.01)	0.68	1994
Immunology and Microbiology	Total^a^	1.32***		(0.02)	0.79	534
	Funded	1.28***	0.08	(0.02)	0.85	424
	Un-funded	1.20***		(0.03)	0.73	533
Neuroscience	Total^a^	1.28***		(0.03)	0.74	578
	Funded	1.30***	0.16	(0.02)	0.88	478
	Un-funded	1.14***		(0.03)	0.63	571
Pharmacology, Toxicology and Pharmaceutics	Total^a^	1.27***		(0.03)	0.68	717
	Funded	1.28***	0.10	(0.02)	0.85	515
	Un-funded	1.18***		(0.03)	0.59	707

*p*-value < 0.001

^a^Total = funded + un-funded; α is the scaling factor, Δ = Difference between α (funded)- *α*(un-funded), SD   Standard Deviation, *R*^2^ is the coefficient of determination, *N*  Number of cases

Table 4 also shows that coefficients of *R*^2^ of the funded articles in all five disciplines of life science are higher than *R*^2^ of the power-law model for un-funded papers, suggesting a better goodness of fit.

To conduct a visual comparison of the Matthew effect based on the power-law correlation between the group of funded and un-funded publications, we classify the results of scaling exponent in three categories according to the intensity of the Matthew effect (Table 5):

- *High* Matthew effect has *α* ≥ 1.29.
- *Medium* Matthew effect has 1.18 < *α* < 1.29.
- *Low* Mathew effect has *α* ≤ 1.18).

**Table 5:** The intensity of Matthew effect power on the number of citations for funded and un-funded publications across research fields of life science

Intensity of power-law scaling effects	Papers *with* funding support	Papers *without* funding support
High Matthew effect		
*α* ≥ 1.29	Agricultural and biological sciences	
Neuroscience		
Medium Matthew effect		
1.18 < *α* < 1.29	Immunology and Microbiology	
Pharmacology, toxicology and pharmaceutics		
Biochemistry, Genetics and Molecular biology	Agricultural and Biological Sciences	
Immunology and Microbiology		
Low Matthew effect		
*α* ≤ 1.18		Biochemistry, genetics and molecular biology
Neuroscience		
Pharmacology, toxicology and pharmaceutics		

*α* is the exponent of power-law correlation. High exponent of scaling for citations suggests information about the strength of Matthew effect in research fields. High *α* indicates a stronger Matthew effect in citation-based performance, vice versa a low value of *α*

### Remark

 The thresholds are calculated using the 25th and 75th percentiles of the distribution of estimated *α* for power-law model (including funded and un-funded data): 25th percentiles (lower) is 1.18; the 75th percentiles (higher) is 1.29.

These results show that funded publications in “Agricultural and Biological Sciences” and “Neuroscience” have a higher Matthew effect compared to other categories, and a funding research policy in such disciplines leads to a higher rate of scaling in citation growth (Table 5). Thus, a research policy implication is that funding these scientific fields of life science can positively impact on scientific performance and knowledge diffusion in society. In general, citations of funded publications in all five disciplines at least scale exponentially at a medium level. Moreover, results suggest that citations of un-funded publications have a medium and lower intensity of Matthew effect; none of them has experienced a high level of Matthew effect in their citation-based performance growth.

Table 6 suggests three levels of Matthew effect between funded and un-funded papers to interpret better the results and recommend more efficient research policy implications considering funding strategies.

**Table 6:** Matthew effect gap between funded and un-funded publications across all five disciplines in life science

Matthew effect gap	Funding leads to *High* Matthew effect	Funding leads to* Medium* Matthew effect	Research Policy implication
Δ ≥ 0.13	Neuroscience		*High priority* in R&D investment: funding leads to a significant increase in citation-based impact, with a higher diffusion of scientific results
0.04 < Δ < 0.13	Pharmacology, Toxicology and Pharmaceutics	Immunology and Microbiology	*Medium priority* in R&D investment: funding generates an average increase in citation-based impact, with an average diffusion of scientific results
Δ ≤ 0.04	Agricultural and Biological Sciences	Biochemistry, Genetics and Molecular Biology	*Low priority* in R&D investment: funding creates a lower increase in citation-based impact, with a lower diffusion of scientific results

*α* is the exponent of power-law correlation, higher exponent of scaling for citations indicates a more powerful Matthew effect; Funding with a High Matthew effect: *α*-funded ≥ 1.29, Funding with a Medium Matthew effect: 1.18 < *α*-funded < 1.29. The information of funding for high/medium Matthew effect is from Table 5. Δ = *α* (funded)- *α* (un-funded) = Matthew effect Gap; higher Δ indicates that funding can have a significant impact on citations compared to un-funded publications. To improve the life science citation-based performance, adequate funding in disciplines with the highest delta (Δ) can generate a great difference in the scientific performance of publications and play an effective role in creating a scaling growth in the number of citations. The thresholds of the first column are calculated using the 25th and 75th percentiles of the values of delta (Δ): 25th percentiles (lower) are 0.04; the 75th percentiles (higher) are 0.13

According to the results here, to conduct an effective policy of research funding to increase the scientific performance of citations of papers in life science, “Neuroscience” with high Matthew effect with funding and high Matthew effect gap (Δ = *α* funded- *α* un-funded) should position in a high priority area for R&D investments in life science. It means that citations of funded publications grow with a high scaling exponent by increasing the number of publications, and this scaling effect is significantly greater than scaling effect of un-funded publications. In “Pharmacology, Toxicology and Pharmaceutics”, since delta (Δ) is at a medium level, investing in this field can also be considered an effective research policy. “Immunology and Microbiology” have both a medium level of Matthew effect gap and Matthew effect power in funded category. Although there is a powerful Matthew effect in funded categories of “Agricultural and Biological Sciences”, citations of both un-funded and funded publications tend to increase regardless of their funding status. Indeed, this deficiency in the performance of funding strategy is greater in “Biochemistry, Genetics and Molecular Biology”: since there is no considerable gap between the scaling exponent of funded and un-funded sets of publications, this research field has a low priority in R&D investment to support citation growth and science diffusion in life science compared to other fields.

**Fig. 2:**
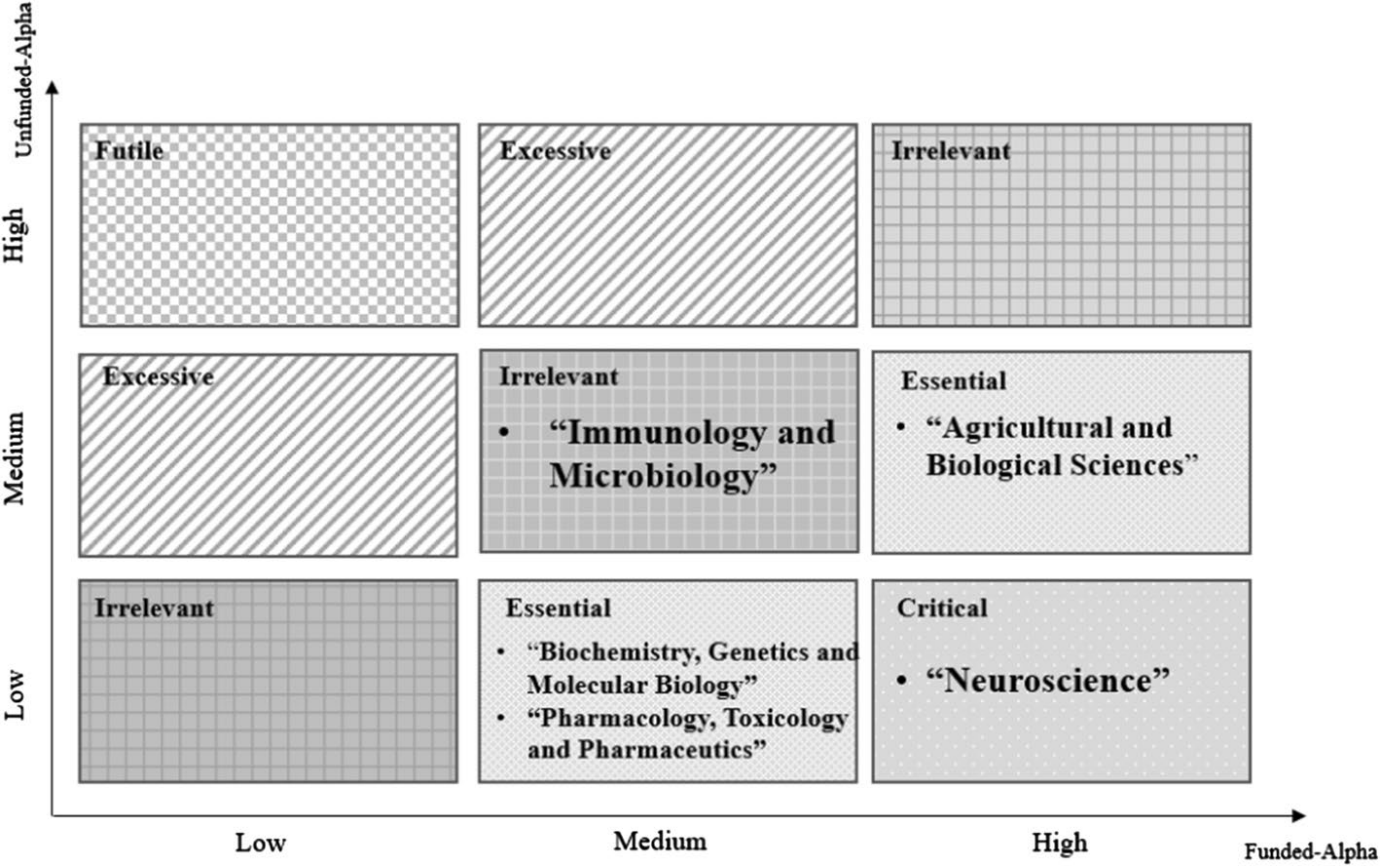
Decision-making matrix for funding policy of research fields in life science according to their location

Finally, the results of Tables 5 and 6 here can be used to suggest a logical scheme represented in Fig. 2 directed to support policymakers in R&D investments. In Fig. 2, the *x*-axis represents the alpha value of funded articles, categorized in Low (*α* ≤ 1.18), Medium (1.18 < *α* < 1.29), and High (*α* ≥ 1.29). The *y*-axis shows the alpha value of un-funded publications with the same categorization: Low, Medium, High. The relationships between alpha of funded and un-funded papers in the scheme of Fig. 2, as explained in methods, are a useful criterion to support funding of research fields considering the strength of Matthew effect in both funded and un-funded publications, as follows:

**Table:** 

*Critical zone*: A high gap between alphas of funded and un-funded articles shows that funding greatly impacts citation growth, and that financial support policy in this field should be a critical and primary aspect for improvement of citation performance, such as in *neuroscience*
*Essential zone*: High funded Matthew effect and medium un-funded Matthew Effect, or medium funded Matthew effect and low un-funded Matthew effect; funding research policy here can boost the citation impact exponentially; moreover, the lower gap between the magnitude of scaling in funded and un-funded categories leads to consider disciplines in this area having a secondary priority of research funding (e.g., *Agricultural and Biological Sciences, Biochemistry, Genetics and Molecular Biology; Pharmacology, Toxicology and Pharmaceutics*)
*Irrelevant zone*: Both funded and un-funded categories are in the same level of Low, Medium, or High; it means that both un-funded and funded publications citation will scale exponentially in favor of the increasing number of publications. Although the magnitude of Matthew effect significantly leads to higher citation performance in High-High and Medium-Medium areas, the gap between funded and un-funded categories is insufficient to support a primary funding research policy for these research fields. Additional factors are necessary to support effective decision-making procedures of R&D funding and investments of research field here—for instance, *Immunology and Microbiology*
*Excessive zone*: funding in this area does not positively impact on citation-based performance, and R&D investment policy for these disciplines may be decreased to have more efficient budgeting
*Futile zone*: funding research policy to support citation performances is unnecessary for research fields in this area. Hence, funding is not an effective strategy in these disciplines to improve scientific performance regarding citations and diffusion of science in society

Figure 2 suggests that a funding policy in “Neuroscience” is critical strategy to improving the scientific impact in life science. Therefore, this field should be supported financially because investing in Neuroscience can greatly improve scientific performance and consequential knowledge diffusion in life science. Furthermore, “Agricultural and Biological Sciences”, “Pharmacology, Toxicology and Pharmaceutics”, and “Biochemistry, Genetics and Molecular Biology” are fields in which funding research policy is an essential strategy for improving the scientific performance of papers and diffusion of scientific research in society. Finally, funding in “Immunology and Microbiology” seems to be an irrelevant research policy that cannot significantly play a role of accelerator for improving citations of papers; here, it is important to consider other relevant factors to make decisions for R&D investments. In short, Fig. 2 can support a research policy of funding in different fields to efficiently increase the citation-based performance of publications in life science for widespread scientific research in science and society.

Finally, these findings, based on case study of life science, can have main theoretical implications to clarify aspects of scientific change and support the research policy of scientific fields, given by following properties (or laws):

- 1st Law. Number of citations of funded and un-funded publications grows through a power-law distribution
- 2nd Law. Funded research has a higher scaling potential than un-funded publications
- 3rd Law. Funded documents receive more citations than un-funded papers in all research fields
- 4th Law. Funded papers have a more substantial cumulative advantage of citations than un-funded papers

### Remark

Funding the research field of Neuroscience increases the citation impact of its publications compared with other fields, whereas funding the research field of Immunology and Microbiology does not significantly affect the citation-based performance of published papers in journals.

## Concluding observations and limitations

This study explores the relationship between research funding and the citation-based performance of scientific output in research fields of life science. The methods focus on scientific output of published papers because: (*a*) scientific output is recorded in on-line databases that are easily accessible, and (*b*) funding status of papers can be detected and in general is provided by agencies after an external revision and assessment that should be and indicator of quality and support a scientific research and output having a high potential impact in science and society (and consequently a high scientific performance). In this context, the study here analyzes the publications recorded in citation database by Scopus (2021) concerning critical disciplines of life science, given by “agricultural and biological sciences”, “biochemistry, genetics and molecular biology”, “Immunology and microbiology”, “neuroscience” and “pharmacology, toxicology and pharmaceutics”. This study does not consider scientific research not published because it is not recorded in datasets, and it is difficult to detect and investigate its funding status due to heterogeneity of content not including all information needed for reliable comparative analyses, such that a scientometric analysis including this research not published may lead to misleading results. In addition, many papers and studies can be funded indirectly by the universities, such as by salary and other benefits that professors and scholars receive from universities and that are not specific resources for doing research, but scholars produce scientific papers for curiosity and other natural characteristics of researchers. Hence, indirect fundings in scientific research are also main aspects but difficult to detect and estimate and including this variable factor can create possible distortions in comparative analyses for assessing the scientific impact, in terms of citation performance, of papers in science and society.

Results here show that although journals publish un-funded articles more than funded publications in life science, funded documents received more citations than un-funded papers. Findings also support that Total (funded + un-funded), funded, and un-funded papers have citations following a power-law distribution in all research fields of life science under study. Our findings confirm previous studies by showing that funding of agencies is a driver to scale the scientific performance of publications, especially citation impact in different disciplines, such as economics, computer science, etc. (Roshani et al., 2021). This main result has been extended here in life science to improve allocation of resources and consequently wellbeing of people and to cope with pandemic crises, such as Coronavirus Disease 2019 (COVID-19), that create socioeconomic problems in society (Coccia, 2021a, 2021b, 2021c, 2021d, 2021e, 2021f). This study is connected to many studies of scientometrics in these topics as described in the section of introduction and theoretical framework (Checchi, 2019; Gök et al., 2015; Reed et al., 2007; Zhao et al., 2018). In fact, some studies investigate the relationship between funding and citation impact with different methodologies showing similar results to analyses discussed here. Yan et al. (2018) analyze the relationship between scientific financing and citation impact in science, technology, engineering, mathematics, and medicine (STEMM) area using a regression model with Heckman bias adjustment and show that financing has a significant relationship with a paper’s citations in STEMM fields. According to another study by Wagner and Jonkers (2017), public R&D investment is only tangentially correlated with the citation impact of a nation’s publications as evaluated by the field-weighted citation index. Leydesdorff et al. (2019) and Benavente et al. (2012) also point out modest coefficients between public funding and citation impact, using a negative binomial regression analysis. Our study here, by employing a different methodology, shows not only a positive relationship between funding and citations of papers in life science but also a cumulative effect of citations over time. Moreover, in this context, a preliminary analysis (to be further developed in future) considers individual data of leading scholars (awarded with Nobel Prize) to assess how funding can affect citations of their papers. In particular, we consider publications and citations of scholars having Nobel Prize in Chemistry and Medicine over 2019–2020 period (Table 7). Results suggest that in Chemistry funded papers have a higher average citation than un-funded papers, vice versa in Medicine.

**Table 7:** Average citations of publications of scholars awarded with Nobel Prize in Chemistry and Medicine in 2019 and 2020

2019–2020	Chemistry		Medicine	
	Funded	Un-funded	Funded	Un-funded
Papers (number)	530	287	1044	641
Citations	95,829	31,063	201,583	144,305
Average citations	180.81	108.23	193.09	225.12

These preliminary results suggest that manifold factors, in addition to institutional funding, can affect the citation and diffusion of knowledge in scientific fields, such as reputation of scholars, nature of research fields, researcher characteristics, institutions and countries in which research is developed, life cycle effects of scholars and papers, etc. In general, the effects of funding on citations of papers provide tentative results, and this study is a starting point for further investigation considering not only data at macro level of research fields, but also data at micro level of leading scholars to analyze the complex factors and dynamics between funding and citations to foster the diffusion of knowledge in science and society.

Although this study has provided some interesting and preliminary results, it has several limitations. First, a limitation of this study is that sources understudy may only capture certain aspects of the ongoing dynamics of citations between research fields in life science. Second, multiple confounding factors could have an essential role in the patterns of citation performance in life science at level of research fields to be further investigated (e.g., institutional aspects, investments, network of collaboration, openness, intellectual property rights, etc.). Third, this study’s computational and statistical analyses focus on data in a specific period and research fields of life science and studies that found the opportunity to be published in journals. Numerous funded and un-funded papers have not been published in journals and deserve to be investigated in future studies with appropriate methods that detect this latent area in science. Finally, future studies can also focus, as said, on individual data of scholars for a benchmark analysis to support current results and clarify aspects and changes of the relation under study at micro and macro level of analysis. Thus, generalizing the results of this research should be done with caution 09.

To conclude, future research should consider more data, and when possible, with new approaches to reinforce proposed findings here (cf., Rodríguez-Navarro & Brito, 2018, 2019). Despite these limitations, the results here clearly illustrate the vital role of funding for a higher scientific impact and diffusion of knowledge in life science but also the need for more detailed examinations of the relationship between patterns of citations and research funding for appropriate research and innovation strategies to foster scientific development to improve the wellbeing of people in society.
